# Development of a rapid, sensitive and visual reverse transcription recombinase-aided amplification coupled with lateral flow dipstick assay for influenza B virus detection

**DOI:** 10.3389/fmicb.2026.1856881

**Published:** 2026-08-04

**Authors:** Yanhong Liu, Heyuan Geng, Yuan Wang, Shanchao Hong, Yue Sun, Qinggui Yang, Shengqiang Wang, Jingdi Pan

**Affiliations:** 1 QT Biotech Co., Ltd, Wuxi, China; 2 Jiangsu International Travel Health Care Center (Nanjing Customs Port Outpatient Department), Nanjing, China; 3 Shaanxi International Travel Healthcare Center (Xi’an Customs Port Outpatient Department), Xi’an, China; 4 Department of Clinical Laboratory, Jiangnan University Medical Center, Wuxi, China; 5 Wuxi Customs, Wuxi, Jiangsu, China; 6 School of Global Health, Chinese Center for Tropical Diseases Research, Shanghai Jiao Tong University School of Medicine, Shanghai, China

**Keywords:** analytical performance, influenza B virus, LFD, point-of-care testing, rapid detection, RT-RAA

## Abstract

**Introduction:**

Influenza B virus (IBV) is a major etiological agent of seasonal influenza, imposing a significant global health burden. Rapid and accurate detection is crucial for epidemic control, but conventional methods are limited by complex equipment or long turnaround times.

**Methods:**

Herein, we developed a reverse transcription recombinase-aided amplification assay combined with lateral flow dipstick (RT-RAA-LFD) for specific IBV detection, targeting the conserved *ns* gene.

**Results:**

Following primer screening, the optimal primer pair F2/R4 was identified. The assay was optimized to react at 39°C for 20 min with a detection volume of 15 μL. Analytical validation demonstrated high sensitivity (50 copies/mL) and no nonspecific cross-reactivity with other common respiratory viruses, including influenza A virus, SARS-CoV-2, parainfluenza virus and adenovirus. Clinical performance evaluation revealed 100% concordance with the gold standard PCR assay.

**Discussion:**

Collectively, the established RT-RAA-LFD assay is rapid, specific, sensitive and easy to operate without relying on sophisticated instruments, making it a promising tool for point-of-care testing (POCT) and field surveillance of IBV infections.

## Introduction

Influenza, a highly contagious respiratory disease caused by the influenza virus, remains a major global health burden. Besides the four terrible pandemics recorded in history, influenza viruses continue to cause seasonal epidemics, resulting in approximately 1 billion infections, 3–5 million severe cases and 290,000–650,000 deaths annually (Din et al., 2023). Influenza B virus (IBV) is one of the primary types responsible for these annual seasonal epidemics. In recent years, it has been reported that the prevalence of IBV strains has increased globally. Notably, IBV has been associated with significantly high mortality rates among children (Centers for Disease Control and Prevention [CDC], 2011; Sharma et al., 2019; Tran et al., 2016). Furthermore, immunocompromised individuals, including HIV-infected patients, children and the elderly, are at high risk of developing severe symptoms, requiring hospitalization or experiencing poor prognosis following IBV infection (Cohen et al., 2013; Han et al., 2024).

At the early stages of infection, it is difficult to distinguish the IBV from other respiratory viruses based on symptoms alone. Establishment of a rapid, simple and accurate diagnosis for IBV will undoubtedly help accelerate the treatment process and control the spread of the virus. Reverse transcription-polymerase chain reaction (RT-PCR), recognized as the gold standard, exhibits high sensitivity (> 95%), excellent specificity and subtype discrimination capability. However, its reliance on sophisticated thermal cyclers and trained personnel, coupled with a long turnaround time, restricts its application in primary healthcare settings or point-of-care testing (POCT) scenarios. Rapid influenza diagnostic tests (RIDTs) based on antigen detection are simple to operate, low-cost and time-saving (15–30 min). Nevertheless, they show relatively low sensitivity (50–70%), which limits their application as a standalone diagnostic tool (Morehouse et al., 2023). Serological assays are not applicable for acute infection diagnosis due to the lag in antibody production (Wang et al., 2026). Virus isolation demonstrates 100% specificity, but it requires specialized biosafety laboratories and a prolonged culture period of 3–10 days. Collectively, these conventional methods present a clear trade-off among sensitivity, speed and operational convenience.

Recombinase-aided amplification (RAA) is a highly appealing isothermal amplification assay (Ngoc and Lee, 2024). The reaction system, containing single-stranded DNA-binding protein (SSB), recombinase and strand-displacing DNA polymerase, can form single-stranded DNA without heating and is completed within 5–30 min at a constant temperature (37–42C°). Combined with reverse transcriptase (RT) and lateral flow dipstick (LFD), RAA assay enables the visualization of the detection results (Ngoc and Lee, 2024; Wei et al., 2025; Zheng et al., 2021). Without the need for a thermal cycler, RAA simplifies the operation process and has attracted great attention as an alternative to conventional PCR, especially at customs, primary healthcare institutions and resource-limited settings (Ghosh et al., 2022; Shen et al., 2021). RAA has been proven to be sensitive, rapid and simple to diagnosis various pathogens, while its application in detecting IBV is relatively rare (Chen et al., 2018; Han et al., 2023; Nie et al., 2022; Tian et al., 2023).

In this study, we have successfully developed a novel RT-RAA detection method for IBV. With high analytical sensitivity and selectivity, the method has been consistent with RT-PCR after testing clinical specimens. The amplicons can be detected with LFD by the naked eye, making the method suitable for IBV diagnosis in settings with scarce medical resource and in emergency situations.

## Materials and methods

### Sample preparation

A total of 49 throat swab samples were collected from asymptomatic inbound travelers at the port. All the samples were verified with two different commercial IBV RT-PCR kits (Daan Gene Co., Ltd., Guangzhou, China; BioGerm Medical Technology Co., Ltd., Shanghai, China) in accordance with the manufacturers’ instructions. Of the 49 samples, 19 tested positive and 30 tested negative.

Other clinical samples positive for the SARS-CoV-2, influenza A virus (IAV) subtype H1N1, IAV subtype H3N2, respiratory syncytial virus (RSV) were preserved in our laboratory. Clinical samples positive for parainfluenza type 1 (PIV **I**), PIV **II**, PIV **III** and adenovirus type 1 (AdV **I**) were provided by QT Biotech Co., Ltd (Wuxi, China). These viruses were used to evaluate the analytical specificity of RT-RAA-LFD assay (*N* = 1, per virus).

IBV pseudovirus was synthesized by Fubio (Suzhou) Biomedical Technology Co., Ltd (Suzhou, China) and was used as reaction template in the following experiments.

The RNA extraction of the above samples was carried out in accordance with the instructions of the nucleic acid extraction reagent (V3-D/R, QT Biotech Co., Ltd., Wuxi, China). After extraction is completed with fully automatic nucleic acid extractor RAA-EX16, nucleic acid products should be stored at -80C°.

### Design of primers and probe

The *ns* gene sequence was downloaded from the GenBank database. Homology analysis was conducted through DNAMAN 7.0 software to screen out highly conserved region as the detection target. Four upstream primers, four downstream primers and one probe were designed based on this sequence. The sequences of primers and probe were shown in Table 1. All the primers and probe were synthesized by Sangon Biotech (Shanghai) Co., Ltd. (Shanghai, China).

**TABLE 1:** Sequences of primers and probe used in the study.

Primers and probe		Sequence (5’–3’)
Primers	F1	CTCAACTCACTCTTCGAGCGTYTYAATGAAGG
	R1	Biotin-GTCTCYCTCTTCTGGTGATAATCGGTGCTCTT
	F2	TCACTCTTCGAGCGTYTYAATGAAGGACAT
	R2	Biotin-TAATTGTCTCYCTCTTCTGGTGATAATCGGTG
	F3	TCTTCGAGCGTYTYAATGAAGGACATYCAA
	R3	Biotin-TCTAATTGTCTCYCTCTTCTGGTGATAATCGG
	F4	AATGAAGGACATYCAAAGCCAATTCGAGCAGC
	R4	Biotin-TAAAGTTCTTCCGTRACCARTCTAATTGTCTC
Probe		Labeled before: CAATTCGAGCAGCTGAAACTGCGGTGGGAGTCTTATCCCAATTTGGTC Labeled after: FAM-CAATTCGAGCAGCTGAAACTGCGGTGGGAGT/THF/TTATCCCAATTTGGTC -C3spacer

Labeled before, unmodified full-length probe sequence; Labeled after, the corresponding modified probe sequence; FAM, 6-carboxyfluorescein; THF, tetrahydrofuran spacer; C3-Spacer, C3 spacer at the 3’ end to block elongation.

To test amplification efficiency, all of the upstream and downstream primers were paired in an orthogonal format. Primer screening was conducted based on amplification curve generated by the reaction. The negative sample was double-distilled water, and the tested samples were the RNA extraction product of pseudovirus of Victoria lineage and Yamagata lineage, two antigenically and genetically distinct lineages of IBVs.

### Establishment of RT-RAA

RT-RAA reaction system was established in accordance with the instructions of the RAA nucleic acid amplification kit (LFD method). RT-RAA basic amplification buffer includes Tris buffer (pH 7.4, 50 mM), potassium acetate (50 mM), polyethylene glycol 20,000, dithiothreitol (7.5 mM), creatine phosphate (30 mM), single-stranded binding protein (800 ng/μL), ATP (7.5 mM), dNTPs (1.5 mM), UvsY protein (50 ng/μL), DNA polymerase (50 ng/μL) and recombinase (10 ng/μL). A total volume of 50 μL reaction system was prepared with 2 μL upstream primers (10 μM), 2 μL downstream primers (10 μM), 0.6 μL of the probe (10 μM), 25 μL of basic amplification buffer, 10.4 μL of double-distilled water, 5 μL of template RNA, and 5 μL of magnesium acetate (10 μM). Then we placed the reaction system in QT-RAA-B6108 constant temperature shaker and briefly pressed the pretreatment key for 4 min. Then the reaction system was incubated at 39 C° for 20 min.

### Establishment of LFD

The universal nucleic acid test strips (Biotin/Fam) (QT Biotech Co,, Ltd., Wuxi, China) were chosen for LFD detection. 10 μL amplification product was mixed with 90 μL PBS solution. The mixed solution was added onto the LFD and incubated for 5 min. Then both the test line (T line) and the control line (C line) show bands, it indicates that the tested samples have the target virus. If only the C line shows a red band, there is no target virus. If no band appears on the C line, the amplification result is invalid.

### Optimization of the RT-RAA-LFD reaction

Optimal reaction conditions, including amplification temperature, amplification time and sample volume of LFD, were determined by comparing the intensity of bands at the T line. The screening process followed a one-variable-at-a-time approach, specifically examining amplification temperatures, amplification time and sample volume. When optimizing the amplification temperature, it is set with gradient of 35, 37, 39, and 41°C. When optimizing the amplification time, it is set with gradient of 5, 10, 20, and 30 min. When optimizing the sample volume, it is set with gradient of 5, 10, and 15 μL. The sample of the negative control (NC) was double-distilled water, and the tested sample was the RNA extraction product of IBV pseudovirus.

### Analytical sensitivity and specificity analysis

The RNA of IBV pseudovirus was serially diluted to 100, 50, 25, and 10 copies /mL, and the analytical sensitivity of the established method was evaluated by the optimized RT-RAA-LFD reaction system. Nucleic acid of IAV subtype H1N1, IAV subtype H3N2, SARS-CoV-2, PIV I, PIV II, PIV III, RSV, and AdV1 was taken as templates, and the analytical specificity of the establishment method was evaluated.

### Evaluation of the integrated density of T line

Analysis of the test line (T line) band intensities was performed using ImageJ (1.54 g/Java 1.8.0_345, 64-bit). A rectangular region was first randomly selected from an empty background area. Background subtraction was carried out by selecting “Process”→“Subtract Background” from the menu bar. Subsequently, each T-line band was outlined using a rectangular selection tool, and its integrated density (IntDen) was measured via the “Measure” command.

### Evaluation of assay performance for clinical samples

To evaluate the performance of the established RT-RAA-LFD assay, 49 throat swab samples were evaluated. The concordance rate between the two assays was calculated with the following formula: (Number of consistent results by both methods/total number) × 100%.

## Results

### Screening the optimal primers

**FIGURE 1:**
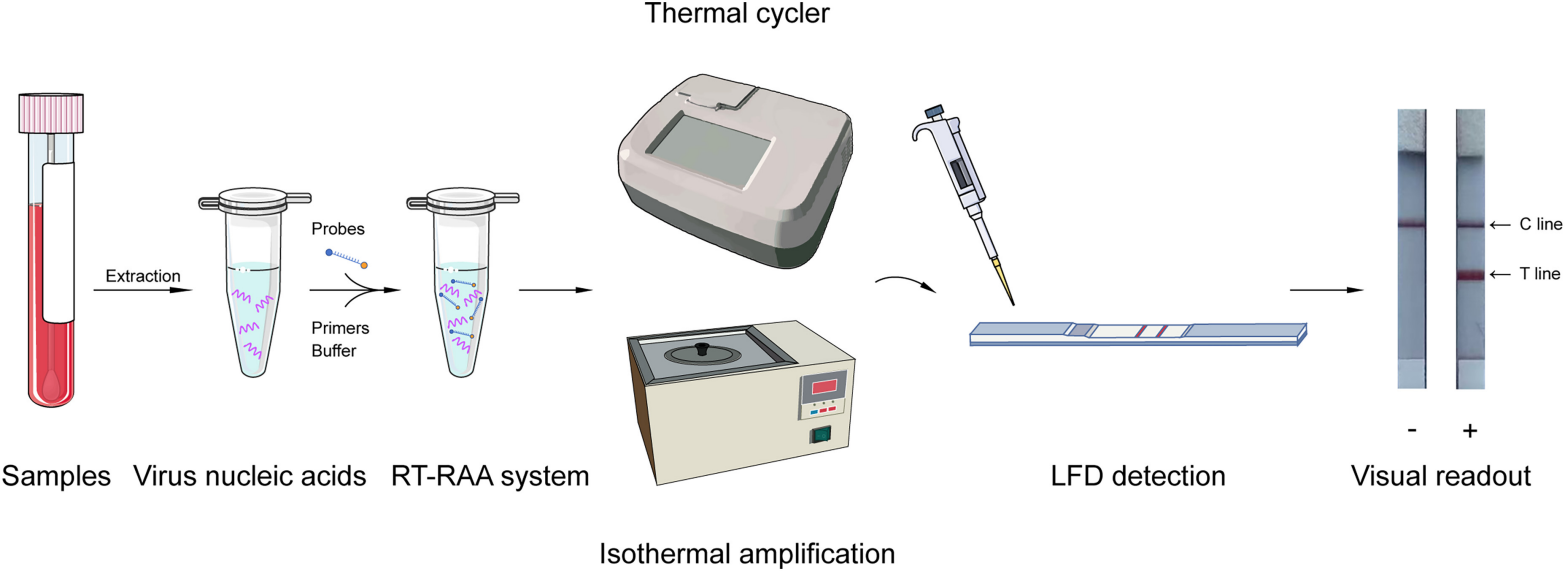
Schematic diagram of the working principle for the RT-RAA-LFD assay in IBV detection. Total RNA was extracted from throat swab samples and added to the RT-RAA reaction mixture. The mixture was then thoroughly blended and incubated at a constant temperature with thermostatic device for nucleic acid amplification. Afterward, the later flow dipstick (LFD) was inserted into the diluted amplification products. Rapid visual readout can be achieved immediately. If both the control (C) line and test (T) line are visible simultaneously, the result is interpreted as positive. If only the C line appears, the result is interpreted as negative.

**FIGURE 2:**
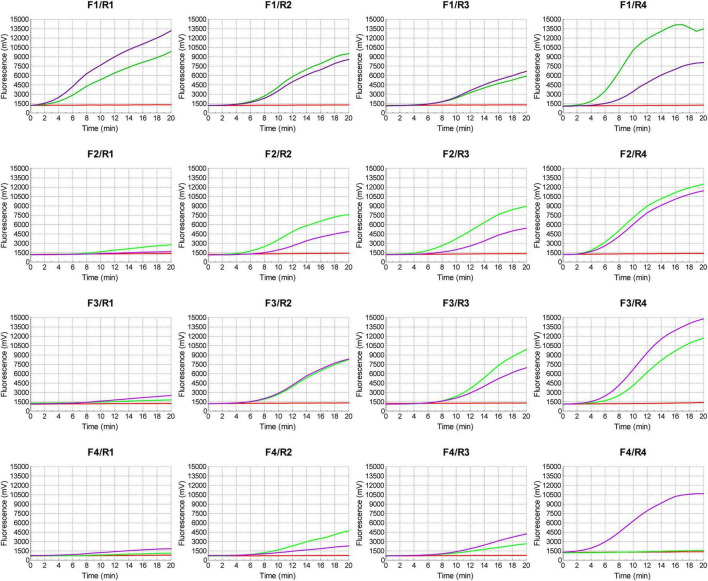
Screening of optical primer pairs for IBV detection. All upstream and downstream primers were combined in an orthogonal manner. The primer pairs were then added to the RT-RAA reaction system and incubated in a constant temperature shaker for amplification. Victoria lineage (Vic) and Yamagata lineage (Yam) were used as templates, represented by the purple and green curves, respectively. NC, double-distilled water (red curve).

As shown in Figure 1, RT-RAA-LFD assay for IBV detection is mainly composed of three parts, including sample preparation, isothermal amplification and results readout with LFD. Firstly, the primers and probes were manually designed based on a 150 bp sequence within the *ns* gene (GenBank Accession No. CY018769.1), which is highly conserved among different IBV strains. Subsequently, four forward primers and four reverse primers underwent RT-RAA screening to identify the most effective combination (Figure 2). During amplification at 39C° for 20 min, 12 primer pairs, including F1/R1, F1/R2, F1/R3, F1/R4, F2/R2, F2/R3, F2/R4, F3/R2, F3/R3, F3/R4, F4/R2, F4/R3, produced positive amplification signals for both Yamagata and Victoria, two antigenically and genetically distinct lineages of IBVs. Among these primer pairs, F2/R4 demonstrated the best performance, characterized by the shortest peak onset time, typical amplification curves for both subtypes and high fluorescence intensity. Therefore, F2/R4 was selected as the optimal primer pair for subsequent experiments.

### Optimization of the RT-RAA-LFD

**FIGURE 3:**
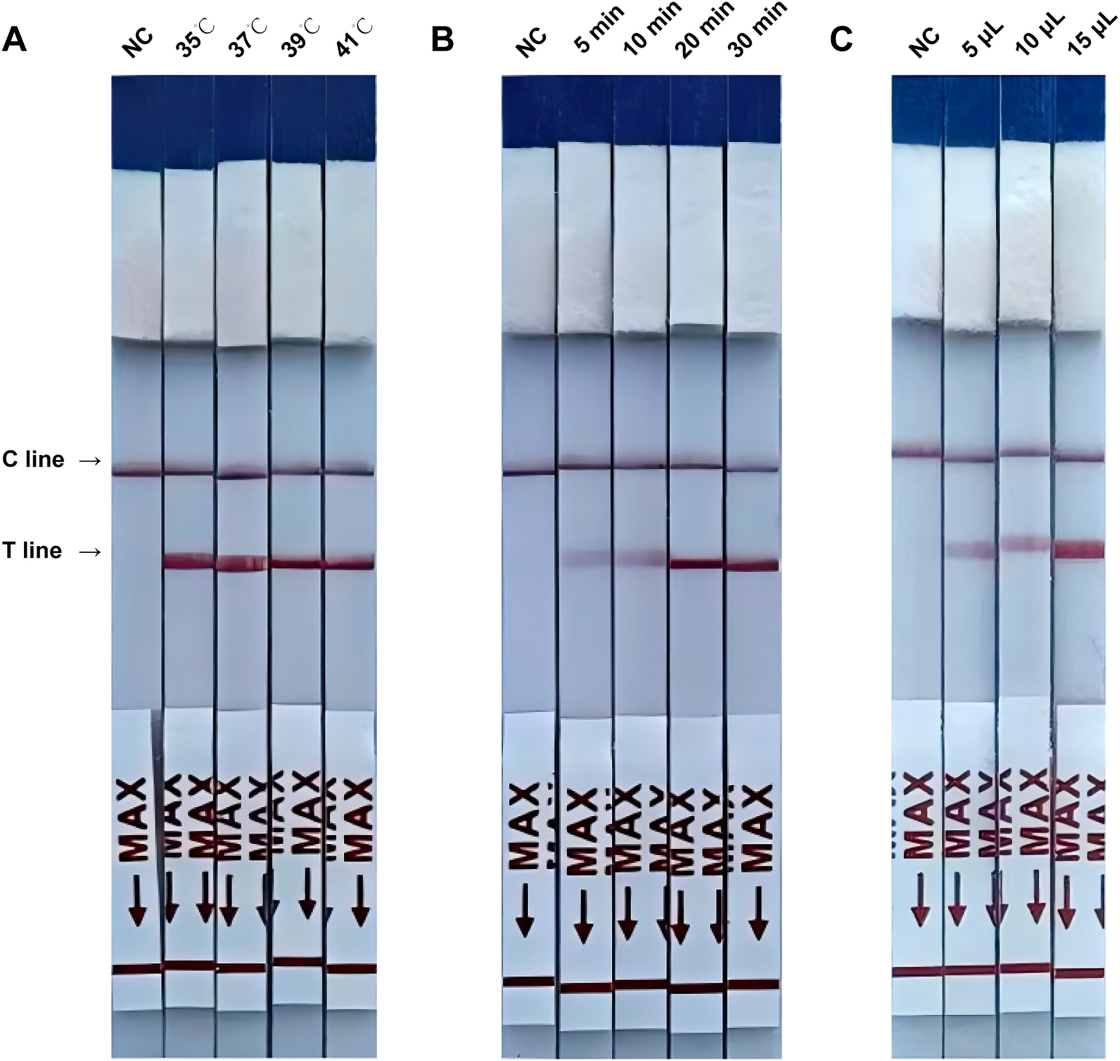
Optimization of the RT-RAA-LFD. Determination of the optical **(A)** amplification temperature, **(B)** amplification time and **(C)** sample volume. NC, double-distilled water. The RNA extraction product of IBV pseudovirus was taken as template for RT-RAA amplification.

To determine the optimal reaction conditions, we evaluated different amplification temperatures, amplification times and sample volumes for the RT-RAA-LFD assay. Quantitative analysis via ImageJ software confirmed comparable band intensities at the test line (T line) across four gradient temperatures (Figure 3A and Table 2). 39°C was selected for subsequent experiments based on the kit’s instructions, which indicate that the recombinase exhibits the highest activity and stability at this temperature. Amplification for as little as 5 min yielded detectable product levels. The intensity of the T line increased gradually with prolonged amplification time and reached saturation at 20 min (Figure 3B). Consequently, 20 min was chosen as the reaction time for further evaluation to ensure high sensitivity for low-concentration templates while minimizing non-specific amplification. Under the optimized conditions of 39°C for 20 min, the intensity of T line band progressively strengthened as the sample volume increased. 15 μL of the RNA template generated the darkest band (Figure 3C). Therefore, a sample volume of 15 μL was selected for the experiments.

**TABLE 2:** The integrated density of T line across the four gradient temperatures.

Amplification temperature	35°C	37°C	39°C	41°C
**IntDen**	2.468	2.599	2.325	2.331

### Analytical sensitivity of the RT-RAA-LFD assay

**FIGURE 4:**
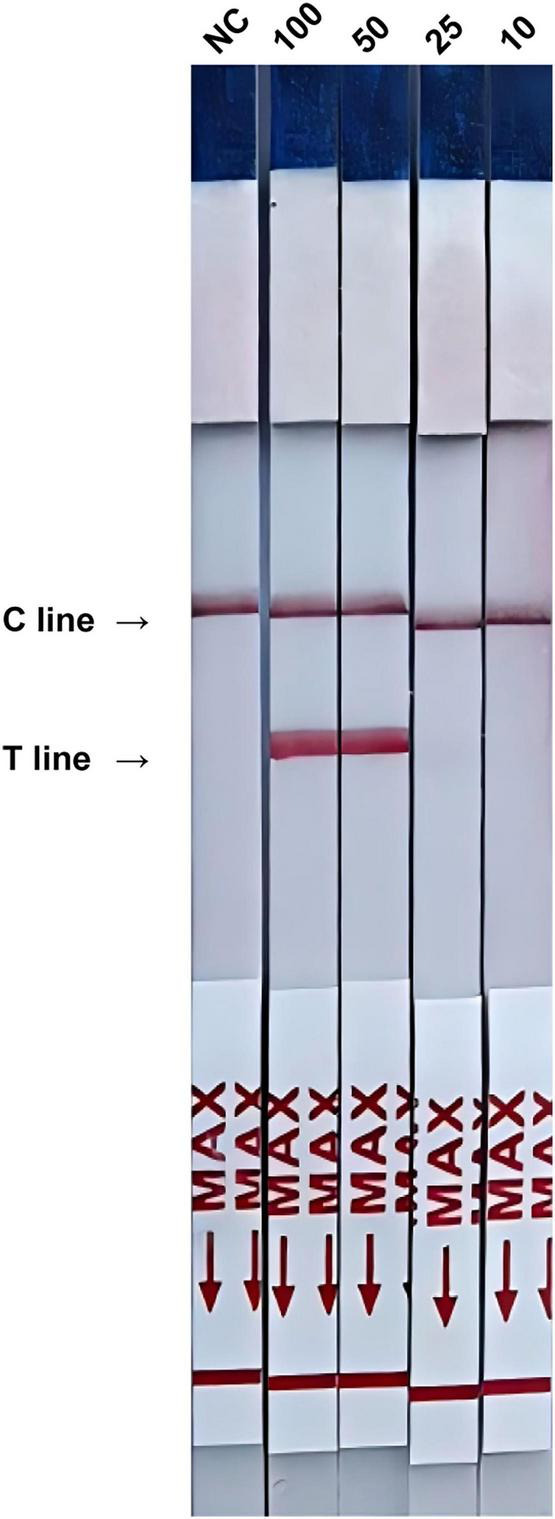
Analytical sensitivity analysis of the RT-RAA-LFD assay. Total RNA extracted from IBV pseudovirus was serially diluted to concentrations of 100, 50, 25, and 10 copies/mL and used as templates in the optimized RT-RAA-LFD reaction system. NC, double-distilled water.

To determine the analytical sensitivity of the RT-RAA-LFD assay, pseudoviruses were used as templates. Total RNA was extracted and serially diluted to concentrations of 100, 50, 25, and 10 copies/mL. As shown in Figure 4, clear T lines and C lines were observed at 100 and 50 copies/mL, implying that the limit of detection (LOD) of the established RT-RAA-LFD assay was 50 copies/mL.

### Analytical specificity of the RT-RAA-LFD assay

**FIGURE 5:**
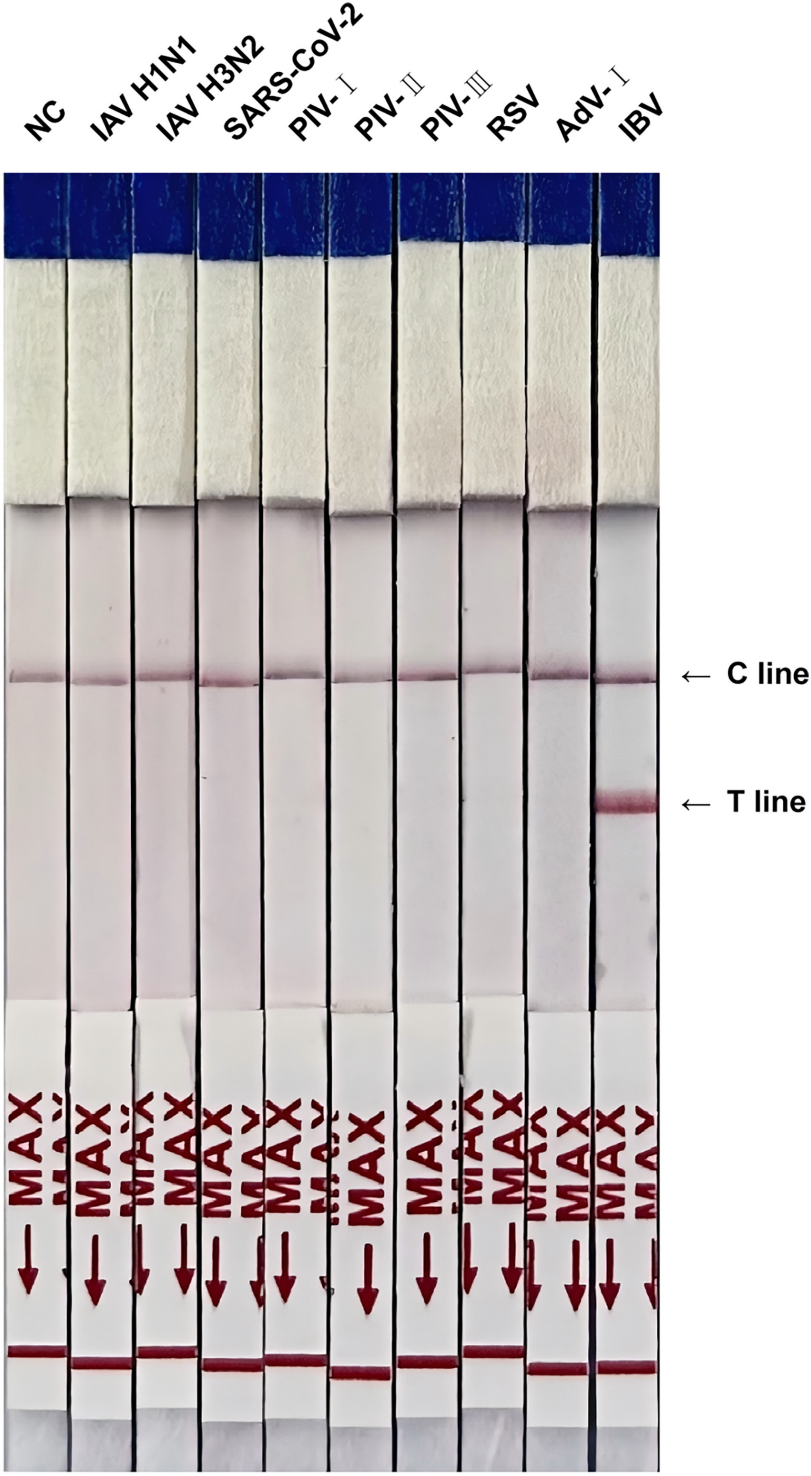
Analytical specificity analysis of the RT-RAA-LFD assay for IBV detection. Nucleic acids from influenza A virus (IAV) subtype H1N1, IAV subtype H3N2, SARS-CoV-2, parainfluenza virus types 1–3 (PIV **I**–**III**), respiratory syncytial virus (RSV) and adenovirus type 1 (AdV **I**) were used as templates in the assay. The nucleic acid of IBV was applied as positive control. NC, double-distilled water.

The analytical specificity of the established RT-RAA-LFD assay was evaluated using a panel of common respiratory viruses as templates. As shown in Figure 5, both of the T and C lines were generated for IBV. In contrast, only C lines were detected for all other respiratory viruses, including influenza A virus (IAV), SARS-CoV-2, parainfluenza (PIV), respiratory syncytial virus (RSV) and adenovirus (AdV). These results confirmed the excellent analytical specificity of the developed assay, which exhibited no cross-reactivity with other common respiratory pathogens.

### Evaluation of the RT-RAA-LFD assay with clinical samples

A total of 49 clinical throat swab samples from inbound personnel at ports, which had been previously confirmed by RT-qPCR (Table 3), were enrolled to assess clinical performance of the RT-RAA-LFD assay for IBV detection. Among these samples, 19 were RT-qPCR-positive (Ct value ranging from 28.37 to 38.73) and 30 were RT-qPCR-negative (Ct > 42). Of the 19 positives, 12 were high Ct samples with values higher than 35 while the remaining 7 were low Ct samples with values < 35. All have shown bands at T line by the RT-RAA-LFD assay, regardless of the Ct values, showing a sensitivity of 100%. For the 30 negatives, all have shown negative results using the RT-RAA-LFD test, providing a specificity of 100%. Collectively, these results demonstrated 100% concordance between the RT-RAA-LFD and the RT-qPCR, indicating that the RT-RAA-LFD assay exhibits robust clinical applicability for IBV detection.

**TABLE 3:** Evaluation of RT-RAA-LFD for detecting clinical samples.

Assay		RT-qPCR		Performance characteristics		
		Positive	Negative	Sensitivity(%)	Specificity(%)	Kappa
RT-RAA-LFD	Positive	19	0	100	100	1
	Negative	0	30			
	Total	19	30			

## Discussion

IAV and IBV are the two most common types responsible for annual seasonal influenza. In previous studies, IBV was generally considered a milder type and has received relatively less attention than IAV (Thompson et al., 2003). However, recent reports have indicated that IBV has increasingly contributed to the annual influenza burden in recent years. In certain seasons, the number of IBV-infected cases can even exceed those caused by IAV (Mao et al., 2025). Notably, IBV has been associated with significantly higher mortality rates, particularly among children (Tran et al., 2016). Therefore, we developed an RT-RAA-LFD based detection assay for rapid differential diagnosis of IBV, which can help prevent treatment delays and control the spread of the virus.

The performance of our newly developed RT-RAA-LFD based assay for IBV detection is highly promising. With a detection limit of 50 copies/mL (equivalent to 0.75 copies/reaction or 0.05 copies/μL), this assay outperforms many previously reported IBV detection methods, such as a CRISPR-Cas13a-integrated RAA assay with LOD of 500 copies/μL, a duplex RPA-LFD assay with LOD of 50 copies/reaction and a multiplex RT-LAMP diagnosis method with LOD of 100 copies/μL (Jang et al., 2020; Sun et al., 2019; Zhang et al., 2025). Additionally, our assay behaves better than the commercial RT-qPCR test kits, like Daan Gene Co., Ltd. and BioGerm Medical Technology Co., Ltd., which have detection limits ranging from 100 copies/mL and 500 copies/mL. Of course, these comparisons are based on LOD values from independent studies conducted under different experimental conditions. Variations in sample types, nucleic acid extraction protocols, analytical instrument, and judgment criteria may impact the comparability of these results. A direct side-by-side comparison using a standardized sample panel under identical operating procedures will be required in future work. Notably, the assay demonstrates excellent analytical specificity, without cross-reactivity observed against common respiratory viruses including IAV, SARS-CoV-2, PIV, RSV and AdV, thereby validating the selection of the conserved *ns* gene as a target. Unlike structural genes (e.g., HA) that are prone to immune pressure-induced mutations, the *ns* gene of influenza B virus is relatively conserved (Andrés et al., 2023; Lindstrom et al., 1999). This feature not only avoids false positives resulting from homology with non-target respiratory pathogens, but also minimizes false negatives caused by viral mutations.

Operating at a constant temperature for just 10 min, the RAA assay developed in this study enables nucleic acid testing without thermal cycling, thereby reducing operational complexity and showing great potential for diverse applications. In clinical practice, it can be utilized as a rapid and accurate diagnostic tool for IBV, particularly in remote regions where PCR facilities are not readily available. Owing to its short reaction time, portability and visual readout via LFD, the assay is well suited for POCT, facilitating immediate diagnosis and treatment decisions at the bedside. In addition, during influenza epidemic seasons, this assay can be employed for large-scale decentralized screening in resource-limited or point-of-care settings to rapidly identify infected individuals and mitigate virus transmission, complementing central laboratory-based high-throughput testing.

Like all diagnostic assays, the developed method presents limitations that warrant further improvement. The small sample size may not fully reflect the performance of the assay in real-world clinical settings. And the cohort lacks sufficient diversity, as 19 positive samples may not comprehensively cover the major circulating IBV lineages and their variants. Positive samples with co-infections of other common respiratory pathogens or with viral loads near the detection limit were limited. Larger-scale clinical trials involving more diverse cohorts will be included to further validate the RT-RAA-LFD assay. Sample pretreatment currently requires nucleic acids extraction. Directly adding sample lysis to reaction buffer may introduce contamination and generate false positive signals. Integration with other system, such as CRISPR-Cas system, likely serves as a potential solution (Cao et al., 2025; Chen et al., 2020; Huang et al., 2021). In this strategy, RT-RAA can rapidly produce abundant specific amplicons with sequences complementary to crRNA. Then crRNA guides Cas to specifically bind the target sequences and the collateral cleavage activity of Cas can be activated. The activated Cas cleaves quenched reporter molecules, converting amplification signals into readable signals. Non-specific amplification products arising from contamination in lysis are not recognized by the crRNA and do not trigger reporter cleavage. This synergistic effect minimizes the interference of sample contaminants and provides a feasible solution to develop one-pot detection assays without nucleic acid extraction. In addition, future work could expand the one-step RT-RAA assay into a multiplex platform for the simultaneous detection of multiple respiratory pathogens, addressing the challenge of differential diagnosis for influenza-like symptoms in clinical practice (Zhou et al., 2025; Zuo et al., 2025).

In conclusion, the RT-RAA-LFD assay targeting the conserved *ns* gene developed in this study has exhibited high analytical sensitivity, excellent specificity, and complete concordance with PCR for the detection of IBV in clinical samples. This assay holds great promise for clinical diagnosis and disease control, particularly in settings where rapid and accurate identification of IBV is critical. Further efforts are warranted to facilitate its broader application in the future.

## Data Availability

The original contributions presented in this study are included in this article/supplementary material, further inquiries can be directed to the corresponding author.

## References

[B1] Andrés C. Del Cuerpo M. Rabella N. Piñana M. Iglesias-Cabezas M. J. González-Sánchez A. et al. (2023). Detection of reassortant influenza B strains from 2004 to 2015 seasons in Barcelona (Catalonia, Spain) by whole genome sequencing. *Virus Res.* 330:199089. 10.1016/j.virusres.2023.199089 37011863 PMC10194390

[B2] Cao Y. Wang J. Fan Z. Xu L. Pan Z. Mo Y. et al. (2025). An extraction-free, lyophilized one-pot RAA-CRISPR assay for point-of-care testing of *Haemophilus influenzae*. *J. Clin. Microbiol.* 63:e0053525. 10.1128/jcm.00535-25 40985812 PMC12607747

[B3] Centers for Disease Control and Prevention [CDC], (2011). Influenza-associated pediatric deaths–United States, September 2010-August 2011. *MMWR Morb. Mortal. Wkly. Rep.* 60 1233–1238. 21918492

[B4] Chen C. Li X. N. Li G. X. Zhao L. Duan S. X. Yan T. F. et al. (2018). Use of a rapid reverse-transcription recombinase aided amplification assay for respiratory syncytial virus detection. *Diagn. Microbiol. Infect. Dis.* 90 90–95. 10.1016/j.diagmicrobio.2017.10.005 29141771

[B5] Chen Y. Mei Y. Zhao X. Jiang X. (2020). Reagents-loaded, automated assay that integrates recombinase-aided amplification and Cas12a nucleic acid detection for a point-of-care test. *Anal. Chem.* 92 14846–14852. 10.1021/acs.analchem.0c03883 33064442

[B6] Cohen C. Moyes J. Tempia S. Groom M. Walaza S. Pretorius M. et al. (2013). Severe influenza-associated respiratory infection in high HIV prevalence setting, South Africa, 2009-2011. *Emerg. Infect. Dis.* 19 1766–1774. 10.3201/eid1911.130546 24209781 PMC3837669

[B7] Din G. U. Hasham K. Amjad M. N. Hu Y. (2023). Natural history of influenza B virus-current knowledge on treatment, resistance and therapeutic options. *Curr. Issues Mol. Biol.* 46 183–199. 10.3390/cimb46010014 38248316 PMC10814056

[B8] Ghosh P. Chowdhury R. Hossain M. E. Hossain F. Miah M. Rashid M. U. et al. (2022). Evaluation of recombinase-based isothermal amplification assays for point-of-need detection of SARS-CoV-2 in resource-limited settings. *Int. J. Infect. Dis.* 114 105–111. 10.1016/j.ijid.2021.11.007 34758392 PMC8572376

[B9] Han J. Yang C. Xiao Y. Li J. Jin N. Li Y. (2024). Influenza B virus: Target and acting mechanism of antiviral drugs. *Microb. Pathog.* 197:107051. 10.1016/j.micpath.2024.107051 39442816

[B10] Han Y. Li F. Yang L. Guo X. Dong X. Niu M. et al. (2023). Imunocapture magnetic beads enhanced and ultrasensitive CRISPR-Cas13a-assisted electrochemical biosensor for rapid detection of SARS-CoV-2. *Biosensors* 13:597. 10.3390/bios13060597 37366962 PMC10296353

[B11] Huang D. Shi Z. Qian J. Bi K. Fang M. Xu Z. (2021). A CRISPR-Cas12a-derived biosensor enabling portable personal glucose meter readout for quantitative detection of SARS-CoV-2. *Biotechnol. Bioeng.* 118 1587–1596. 10.1002/bit.27673 33410130

[B12] Jang W. S. Lim D. H. Nam J. Mihn D. C. Sung H. W. Lim C. S. et al. (2020). Development of a multiplex isothermal amplification molecular diagnosis method for on-site diagnosis of influenza. *PLoS One* 15:e0238615. 10.1371/journal.pone.0238615 32915821 PMC7485819

[B13] Lindstrom S. E. Hiromoto Y. Nishimura H. Saito T. Nerome R. Nerome K. (1999). Comparative analysis of evolutionary mechanisms of the hemagglutinin and three internal protein genes of influenza B virus: Multiple cocirculating lineages and frequent reassortment of the NP, M, and NS genes. *J. Virol.* 73 4413–4426. 10.1128/JVI.73.5.4413-4426.1999 10196339 PMC104222

[B14] Mao J. Chu K. B. Eom G. D. Yoon K. W. Heo S. I. Kang H. J. et al. (2025). Influenza A hemagglutinin virus-like particles confer protection against influenza B virus infection. *Emerg. Microbes Infect.* 14:2494702. 10.1080/22221751.2025.2494702 40230200 PMC12039411

[B15] Morehouse Z. P. Chance N. Ryan G. L. Proctor C. M. Nash R. J. (2023). A narrative review of nine commercial point of care influenza tests: An overview of methods, benefits, and drawbacks to rapid influenza diagnostic testing. *J. Osteopath. Med.* 123 39–47. 10.1515/jom-2022-0065 35977624

[B16] Ngoc L. T. N. Lee Y. C. (2024). Current trends in RNA virus detection via nucleic acid isothermal amplification-based platforms. *Biosensors* 14:97. 10.3390/bios14020097 38392016 PMC10886876

[B17] Nie M. Zhou Y. Li F. Deng H. Zhao M. Huang Y. et al. (2022). Epidemiological investigation of swine Japanese encephalitis virus based on RT-RAA detection method. *Sci. Rep.* 12:9392. 10.1038/s41598-022-13604-4 35672440 PMC9172605

[B18] Sharma L. Rebaza A. Dela Cruz C. S. (2019). When “B” becomes “A”: The emerging threat of influenza B virus. *Eur. Respir. J.* 54:1901325. 10.1183/13993003.01325-2019 31416813

[B19] Shen X. Wang J. Li J. He A. Liu H. Ma X. (2021). Field validation of a rapid recombinase aided amplification assay for SARS-CoV-2 RNA at customs - Zhejiang Province, China, January 2021. *China CDC Wkly.* 3 973–976. 10.46234/ccdcw2021.236 34804630 PMC8598545

[B20] Sun N. Wang Y. Yao X. Chen F. Gao D. Wang W. et al. (2019). Visual signal generation for the detection of influenza viruses by duplex recombinase polymerase amplification with lateral flow dipsticks. *Anal. Bioanal. Chem.* 411 3591–3602. 10.1007/s00216-019-01840-z 31079175

[B21] Thompson W. W. Shay D. K. Weintraub E. Brammer L. Cox N. Anderson L. J. et al. (2003). Mortality associated with influenza and respiratory syncytial virus in the United States. *JAMA* 289 179–186. 10.1001/jama.289.2.179 12517228

[B22] Tian Y. Fan Z. Zhang X. Xu L. Cao Y. Pan Z. et al. (2023). CRISPR/Cas13a-Assisted accurate and portable hepatitis D virus RNA detection. *Emerg. Microbes Infect.* 12:2276337. 10.1080/22221751.2023.2276337 37882492 PMC10796118

[B23] Tran D. Vaudry W. Moore D. Bettinger J. A. Halperin S. A. Scheifele D. W. et al. (2016). Hospitalization for influenza A versus B. *Pediatrics* 138:e20154643. 10.1542/peds.2015-4643 27535144

[B24] Wang B. Wei Y. Dong X. Lu Y. Deng S. Chen C. (2026). Comparative evaluation of RNA isothermal amplification-gold probe lateral flow assay and targeted next-generation sequencing for the detection of *Mycoplasma pneumoniae* and influenza A and B viruses in children. *Front. Pediatr.* 14:1741420. 10.3389/fped.2026.1741420 41867928 PMC12999799

[B25] Wei B. Wang W. Guo Z. Yin W. Cheng M. Yang Y. et al. (2025). Rapid visual detection of hepatitis E virus combining reverse transcription recombinase-aided amplification with lateral flow dipstick and real-time fluorescence. *J. Clin. Microbiol.* 63:e0106424. 10.1128/jcm.01064-24 39817756 PMC11837526

[B26] Zhang X. Chen S. Li J. Liu D. A. Lai J. Song X. et al. (2025). One-step RAA and CRISPR-Cas13a method for detecting influenza B virus. *Microb. Biotechnol.* 18:e70144. 10.1111/1751-7915.70144 40231967 PMC11998173

[B27] Zheng Y. Z. Chen J. T. Li J. Wu X. J. Wen J. Z. Liu X. Z. et al. (2021). Reverse transcription recombinase-aided amplification assay with lateral flow dipstick assay for rapid detection of 2019 novel coronavirus. *Front. Cell. Infect. Microbiol.* 11:613304. 10.3389/fcimb.2021.613304 33598439 PMC7882697

[B28] Zhou Y. Liang C. Long Z. Fan L. Wang Y. Wang Z. et al. (2025). Multiplex real-time reverse transcription recombinase-aided amplification assay for the detection of SARS-CoV-2, influenza A virus, and respiratory syncytial virus. *Microbiol. Spectr.* 13:e0275924. 10.1128/spectrum.02759-24 40323293 PMC12131773

[B29] Zuo W. Shao C. Qin H. Gao H. Sun X. Wang P. et al. (2025). Development of a dual-target RAA-LFD assay for point-of-care and visual detection of *Salmonella* pullorum and *Salmonella* typhimurium in fecal samples. *Front. Vet. Sci.* 12:1684537. 10.3389/fvets.2025.1684537 41255765 PMC12621324

